# Understanding health system resilience in responding to COVID-19 pandemic: experiences and lessons from an evolving context of federalization in Nepal

**DOI:** 10.1186/s12913-024-10755-0

**Published:** 2024-04-04

**Authors:** Shophika Regmi, Maria Paola Bertone, Prabita Shrestha, Suprich Sapkota, Abriti Arjyal, Tim Martineau, Joanna Raven, Sophie Witter, Sushil Baral

**Affiliations:** 1 HERD International, Kathmandu, Nepal; 2 Institute for Global Health and Development, Queen Margaret University, Edinburgh, UK; 3 School of Public Health, University of Alberta, Alberta, Canada; 4 Liverpool School of Tropical Medicine, Liverpool, UK

**Keywords:** Health system, Health policy, Resilience, COVID-19, Decentralization, Federalization

## Abstract

**Introduction:**

The COVID-19 pandemic has tested the resilience capacities of health systems worldwide and highlighted the need to understand the concept, pathways, and elements of resilience in different country contexts. In this study, we assessed the health system response to COVID-19 in Nepal and examined the processes of policy formulation, communication, and implementation at the three tiers of government, including the dynamic interactions between tiers. Nepal was experiencing the early stages of federalization reform when COVID-19 pandemic hit the country, and clarity in roles and capacity to implement functions were the prevailing challenges, especially among the subnational governments.

**Methods:**

We adopted a cross-sectional exploratory design, using mixed methods. We conducted a desk-based review of all policy documents introduced in response to COVID-19 from January to December 2020, and collected qualitative data through 22 key informant interviews at three tiers of government, during January-March 2021. Two municipalities were purposively selected for data collection in Lumbini province. Our analysis is based on a resilience framework that has been developed by our research project, ReBUILD for Resilience, which helps to understand pathways to health system resilience through absorption, adaptation and transformation.

**Results:**

In the newly established federal structure, the existing emergency response structure and plans were utilized, which were yet to be tested in the decentralized system. The federal government effectively led the policy formulation process, but with minimal engagement of sub-national governments. Local governments could not demonstrate resilience capacities due to the novelty of the federal system and their consequent lack of experience, confusion on roles, insufficient management capacity and governance structures at local level, which was further aggravated by the limited availability of human, technical and financial resources.

**Conclusions:**

The study findings emphasize the importance of strong and flexible governance structures and strengthened capacity of subnational governments to effectively manage pandemics. The study elaborates on the key areas and pathways that contribute to the resilience capacities of health systems from the experience of Nepal. We draw out lessons that can be applied to other fragile and shock-prone settings.

**Supplementary Information:**

The online version contains supplementary material available at 10.1186/s12913-024-10755-0.

## Introduction

Resilience has emerged as a key concept for health systems in the last decade. Catastrophic events such as economic crises, infectious disease outbreaks, civil unrest, and other shocks have highlighted the need to understand the concept of resilience in relation to health systems and reflect on how to effectively build resilient health system to cope with shocks and crises [1]. The relevance of resilience in relation to health systems was further highlighted globally during the 2020–2021 pandemic of coronavirus disease (COVID-19). The concept and definition of resilience is evolving and gaining greater interest and attention. Blanchet et al. defined resilience as the capacity of a health system to prepare and respond to shocks and to adapt and transform to cope with those, while ensuring delivery of quality and essential health services [2, 3]. Recent literature has highlighted that resilience does not always imply a strong health system with the view that health systems can be strong in stable conditions but may prove vulnerable to shocks or, a health system can be resilient during emergencies but not performing well in routine conditions [4]. COVID-19 has tested the resilience capacities of health systems worldwide to respond to the pandemic while maintaining routine health functions. While it is agreed that the ability of the health system to deal with such shocks and remain resilient depends on the governance and political economy of the local context [5], more research is needed to explore the elements and pathways (which we call “resilience capacities”) that can support heath system resilience during shocks and crises, and generalise lessons learned from case studies. This study explores the health system response to COVID-19 in the new federal context of Nepal and examines the processes of policy formulation, policy communication and implementation at the three tiers of government, including the dynamic interactions between these tiers. It also reflects on how these processes might have affected the response and the longer-term resilience capacities of the health system as well as the role played by resilience capacities of the health system in allowing effective or suboptimal absorption, adaptation, and transformation in the face of a shock.

**Fig. 1:**
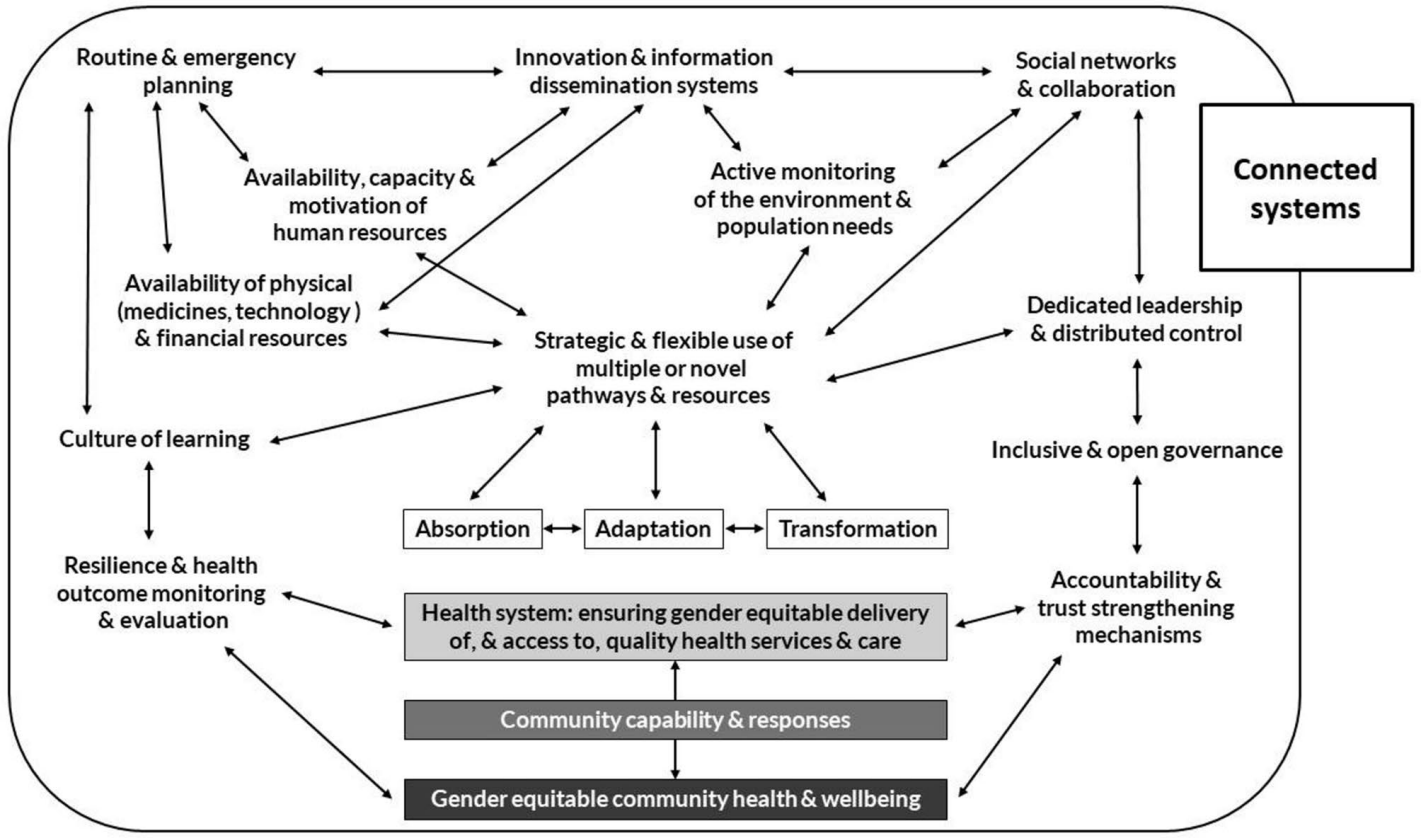
Resilience framework

To guide our understanding of health system resilience and identify core capacities that underlie resilience which may have been activated and/or supported during the COVID-19 response, we adopted the resilience framework and hypotheses developed by ReBUILD for Resilience in 2020 [6] (Fig. 1), based on earlier literature on the topic, including that undertaken by team members (e.g. Jamal et al. in the analysis of health system resilience in Syria) [7]. Under this framework, the broader capacities that the health system must have in place in order to deploy resilience approaches are depicted as enabling the core (absorption, adaptation and transformation) approaches. Resilience capacities refer both to specific elements, such as the presence of a culture of learning within the health system, as well as the pathways, strategies and interlinkages between capacities that reinforce each other (for example, the framework hypothesises that effectiveness of learning processes is related to inclusivity and open governance and decision-making) [6].

### Study setting

In Nepal, a new constitution was promulgated in September 2015, replacing the unitary government and declaring the country as a federal democratic republic comprising of three autonomous governance levels: the federal, the province (7 provinces) and the local level (with 753 municipalities). The introduction of this federalised structure was still at an early stage when COVID-19 pandemic hit the country. Municipalities in the new structure are responsible for the delivery of basic health services in addition to other functions related to formulation of local plans and implementation of health programmes. The functions and responsibilities across the three tiers of governments were defined, however clarity in terms of implementation of these functions and the existence of capacity gaps especially at municipality level were already known before the pandemic [8, 9]. The chronic stress due to the decentralization processes with the added acute crisis due to the COVID-19 pandemic posed a major challenge for the local governments that had to prepare and respond to the pandemic, while at the same time keeping basic healthcare delivery intact, which they were only starting to manage and oversee directly.

**Fig. 2:**
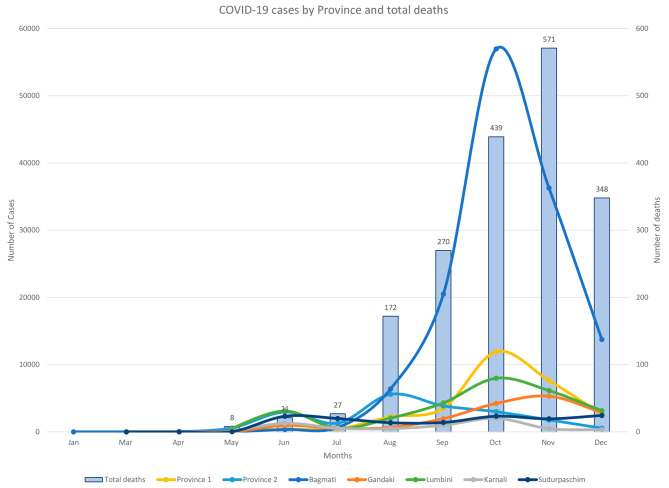
Distribution of COVID-19 cases by province and total deaths from January to December 2020

Nepal was heavily affected by the COVID-19 pandemic during the first wave in 2020. Figure 2 presents the distribution of COVID-19 cases from January– December 2020 by province in Nepal, including Bagmati province where the capital city Kathmandu lies, which reported the highest number of cases [10]. However, it should be noted that data also reflect changing government guidelines on testing (for example, from June 2020 no tests were required for asymptomatic cases in quarantine). The government’s response to COVID-19 started in January 2020, and a first lockdown was imposed between March and July 2020, though it was already partially lifted in late May. A much higher wave of cases occurred from August onwards, due to the influx of Nepali migrant workers returning from India and the time of the festivals of *Dashain, Tihar* and *Chhath* in October, during which the government had relaxed restriction on transportation.

## Methods

This study adopted a cross-sectional design using a mix of policy review and primary data from key informant interviews (KIIs). With the aim of understanding the COVID-19 policy response mechanisms adopted by the federal, provincial and local governments to implement basic health services along with preparedness and response to COVID-19, we selected the study sites from all three tiers of the government - Kathmandu (federal level), Lumbini Province (provincial level) and two municipalities of Kapilvastu district (municipal/local level) which was one of the districts with highest COVID-19 cases, bordering with India.

We first conducted a desk-based review of health sector policies, guidelines, and directives on COVID-19 preparedness and response formulated at national and subnational levels from January to December 2020. Document search was carried out mostly online with frequent and regular visits to governmental websites that provide all the COVID-19 policies, guidelines, directives and other relevant documents (Table 1). Out of a total of 90 policies and other guiding documents and directions published by the government over the year 2020 regarding COVID-19 preparedness and response, 76 policies were identified as most relevant and data extracted from them.

**Table 1:** Sources of documents for policy review

Sources of documents
1. Government of Nepal, Ministry of Health and Population (MoHP), Department of Health Services (DoHS), Epidemiology and Disease Control Division (EDCD)
2. Government of Nepal, MoHP, Health Emergency and Disaster Management Unit and Health Emergency Operation Center (HEOC)
3. Government of Nepal, Ministry of Federal Affairs & General Administration (MoFAGA)
4. Government of Nepal, MoHP, DoHS, National Public Health Laboratory
5. Government of Nepal, Ministry of Home Affairs
6. Public Health Update

Secondly, we carried out KIIs in January-March 2021, at federal and sub-national levels to complement and triangulate the information from the policy review, in order to better understand the process of policy formulation, communication and implementation at all levels. In total, 22 KIIs were conducted with participants from federal, provincial and local levels, purposively selected considering their roles in COVID-19 response (Table 2). The interviews took place in Nepali language and lasted for 60 to 90 min. Topic guide was developed in English and translated into Nepali prior to data collection and was further revised and adapted iteratively based on the field experiences (topic guide in Supplementary File 1). KIIs were audio-recorded after receiving consent from the key informants, and then transcribed and translated into English for analysis.

**Table 2:** Summary of key informant interviews

Level and total KIIs	Informants	No. of informants	
		Mun1	Mun2
**Local** ** (16) — 2 municipalities**	Health workers	3 (1 F,2 M)	3 (1 F,2 M)
	Municipality Health Coordinators	1 (M)	1 (M)
	Mayor	1 (M)	1 (M)
	Ward chairs	1 (M)	1 (M)
	Female Community Health Volunteers (FCHVs)	2 (F)	2 (F)
**Province (4)—**Lumbini Province	Provincial Ministry of Health official	1 (M)	
	Provincial Health Directorate (PHD) official	1 (M)	
	External Development Partner (EDP) representative	1 (M)	
	District hospital	1 (M)	
**Federal (2)**	MoHP official	1 (M)	
	EDP representative	1 (M)	
**Total**		**22 (6 F, 16 M)**	

Note: Mun1 = Municipality 1, Mun2 = Municipality 2; F = female, M = male

All data from the document review and KIIs were extracted to provide a descriptive overview and timeline of the policy formulation processes, as well as the policy communication and implementation. At a more analytical level, data analysis took a thematic framework analysis approach and was based on a list of themes and subthemes derived from components of the resilience framework [6], and emerging themes from the data, in line with study objectives. Data was coded using a qualitative software NVivo and was processed iteratively with regular discussion among research team members. Thorough triangulation of information from policy review and KIIs was also carried out, and data was then summarized and organized under defined themes and sub-themes.

## Results

Findings from the policy review are presented in terms of the trajectory of COVID-19 related policy documents over a one-year period. Moreover, data from policy review and KIIs are analysed and presented using a structure of the broader thematic order aligned with the components of resilience framework and supported by quotes extracted from the original transcripts.

### Emergency planning and policy development

#### COVID-19 governance structure and key actors in the policy formulation process

**Fig. 3:**
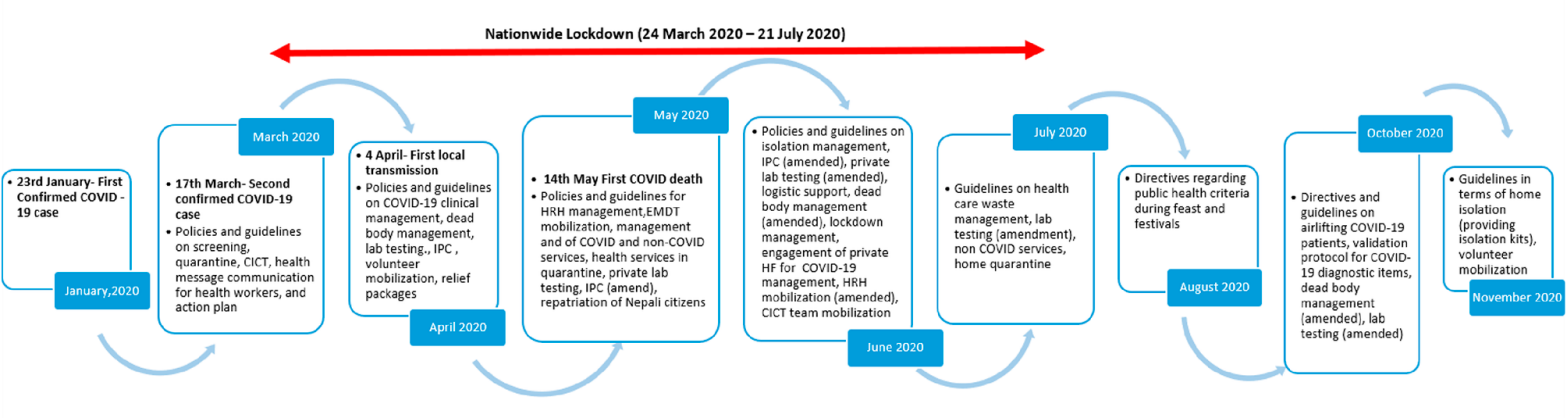
Timeframe of COVID-19 policies and guidelines. *Note*: Abbreviations used in the Fig. 3 - CICT (Case Investigation and Contact Tracing), IPC (Infection Prevention and Control), HRH (Human Resource for Health), HF (Health Facility), EMDT (Emergency Medical Deployment Team)

After the confirmation of first COVID-19 case on 23rd January 2020 in Nepal, the Government of Nepal started formulating various policies and directions in response to COVID-19 pandemic starting from March 2020. Figure 3 below provides an overview of the timing of main events and policies that took place or were published in the period between January and December 2020.

**Fig. 4:**
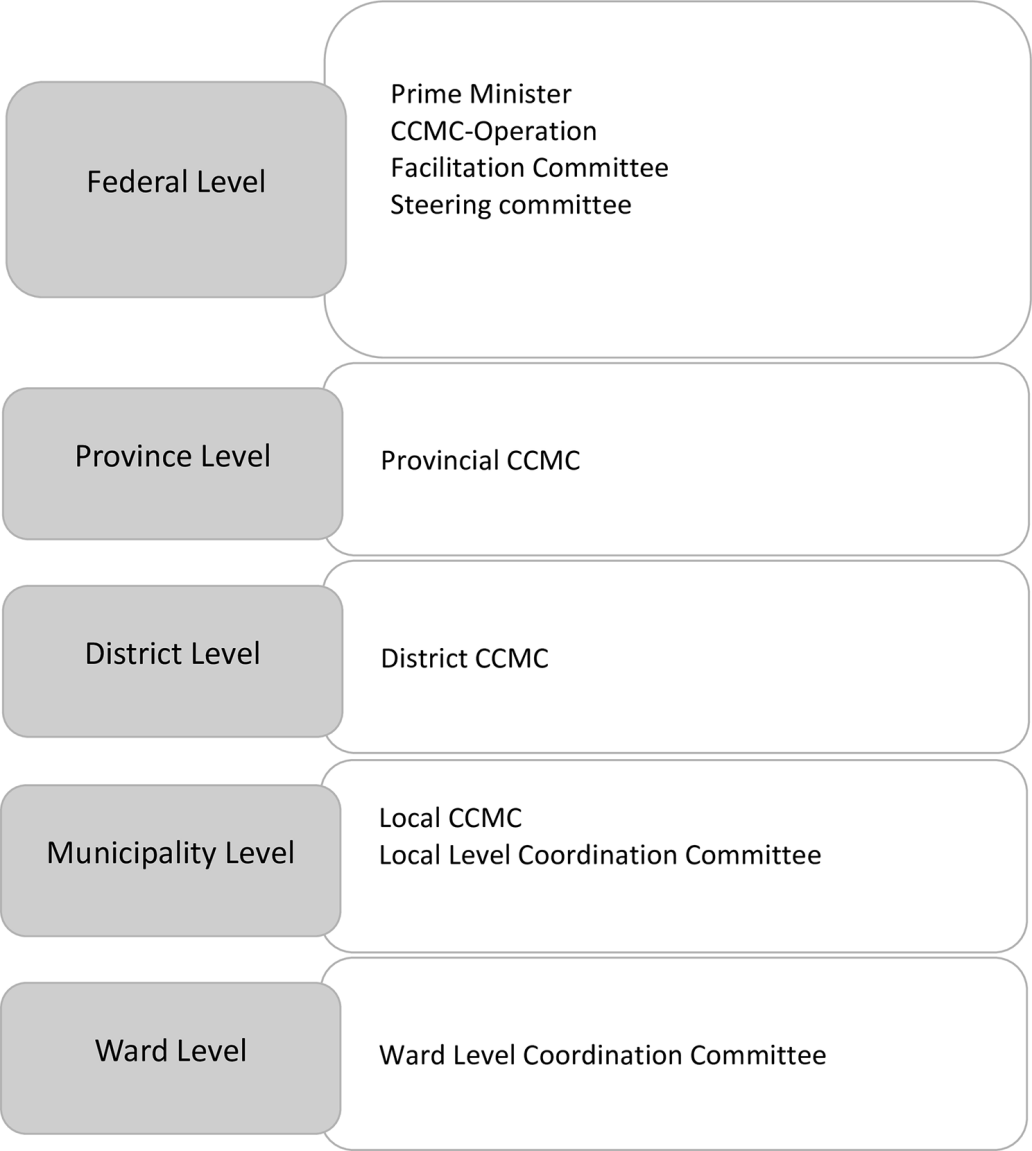
COVID-19 management and response structure

The policy formulation process followed an already existing governance structure with different committees and working groups at national and sub-national levels, and involved engagement of different tiers of government as well as across government agencies and sectors (shown in Fig. 4). After the first COVID-19 case was diagnosed in Nepal in January 2020, the Government of Nepal formed a High-Level Coordination Committee, led by the Prime Minister and the Minister of Defence. Soon after COVID-19 was declared a pandemic on March 11, 2020 by the WHO, [11] three different committees– the Direction Committee, the Facilitation Committee, and the COVID-19 Crisis Management Centre (CCMC) were formed for rapid and integrated response for the prevention and management of COVID-19. The Council of Ministers formed the CCMC primarily responsible for managing the responses in an integrated manner, through its representative units at province, district and local levels [12].

Likewise, the Incident Command System run under the MoHP, led by MoHP’s Secretary, was primarily responsible for developing and refining policies and guidelines for COVID-19 management, works in three different areas– coordination and monitoring, operation and information/data. Later in May, for conducting contract tracing effectively, the MoHP issued a directive for formation and mobilization of Case Investigation and Contract Tracing Teams (CICTT) in each local government [12]. Likewise, the MoFAGA issued a directive for the formation of the local level coordination committee and ward level coordination committee for mobilizing health workers and FCHVs, ensuring health message communication in accordance with MoHP guidelines, providing suggestions and establishing immediate referral systems, monitoring health desks at border entry points, ensuring self-quarantine and physical distancing, etc. [13, 14]. However, in our synthesis, we found that community participation in, and the functionality of these coordination committees was a challenge which raises concerns about whether the planning and policy formulation process was inclusive and participatory.

#### Participation of subnational government in policy formulation (vertical collaboration)

With the federalization of the country, the province and local governments have power to make their own local policies and plans. At the same time, pandemic or any emergency management falls under the prime responsibility of the federal government [15]. Therefore, considering the emergency situation and the limited time available to respond to the pandemic, the federal government effectively led the overall policy development process with little or no consultation with province and local governments, which engaged mostly in implementation of policies and directions for COVID-19 management (for example, quarantine management). Although there were some exceptions (for example, the HEOC meetings, which included province level representatives), most respondents at provincial government level felt they were insufficiently involved in the policy formulation processes.

*Province [government] was less involved in the policy formulation process at federal level. Some draft documents were shared [with province] to collect feedback but nobody has time to review those documents and hence, finalized [policies and guidelines] were sent at once, whereas some documents were developed and circulated without our concern.* (EDP_ Province).

There was no inclusion of local level representatives such as mayors, deputy mayors, executive officers, health coordinators and chiefs of health offices in the formulation of COVID-19 related policies and documents at the province level, who were involved in routine health policies formulation process in the province in the non-COVID context.

#### Multi-sectoral collaboration and networks

Multi-sectoral collaboration was widely observed in federal and provincial levels during the policy formulation process. Participation from different ministries like Ministry of Foreign Affairs and General Administration, Ministry of Home Affairs, Ministry of Industry, Commerce and Supplies, Ministry of Communication and Information Technology, as well as medical associations, security forces (Nepal Army, Armed Police Force) was reported in policy formulation and response activities. Although there was a delay in decision from the government to involve the private sector in the COVID-19 response, from June 2020, the federal government engaged with the private health sector for testing and treatment through a reimbursement mechanism [16]. In addition, consultation with partners, such as the World Health Organization (WHO), international non-government organizations (NGOs) and technical experts were regularly held during policy formulation processes at federal level.

Likewise, province government also coordinated and collaborated with other departments and ministries and also with international NGOs and private sectors, WHO, United Nations Children’s Fund (UNICEF), United Nations Population Fund, Red Cross Society, representatives from medical colleges and Association of Private Health Institution of Nepal, Nepal Commission Drug Association and other local organizations for technical assistance while developing policies. Nevertheless, community level representation was missing in both federal and provincial policy formulation processes.

*While formulating the policies, local problems and needs have to be addressed. The policies are developed at the national level, but they do not align with our local context. Our local level is not developed enough to implement the policies due to many difficulties such as lack of human resources, finance. (Mayor_Municipality2)*

#### Strategic use of evidence

The federal government successfully used global evidence in policies and guidelines developed at federal level, despite the lack of a dedicated professional team and mechanisms for local evidence generation. For example, it considered WHO interim recommendations in different areas for COVID-19 management, and regularly adapted them to some extent while developing national policies and guidelines. Similarly, the province level considered federal policies and WHO technical guidelines. However, during the policy formulation at the federal and provincial levels, identification of local resource needs for example in terms of health staff, logistics, health infrastructure, etc. was found to be done on an ad-hoc basis using assumptions, rather than based on local information and monitoring of local environment and population needs and outcomes.

*We developed a concept about how to treat if there are 5000 critical cases in Lumbini province. Consequently, we formulated a plan including how many HR and equipment will be required, etc. Thereafter, we made a contingency plan assuming how to treat if there are 5000 cases. We made an action plan accordingly to manage [COVID-19] for six months as we were unknown about how long will [COVID 19] last for. (Province official).*

#### Applicability and relevance of national policies to local context

Local level respondents clearly felt the inapplicability and often the irrelevance of national level policies at local level as COVID-19 response and policies were not developed considering local context and lacked coordination with local levels. For instance, differences between urban and rural areas were evident in terms of infrastructure and human and financial capacity, but the same policy was applied to both settings.

*I did not find the national policy to be appropriate to local context. Moreover, I felt that national COVID-19 policy was promoting the autocratic style of enforcing the activities. (Ward Chair_Municipality1)*

This was reiterated by development partners in the province who stated that federal policies were vague and too general. For instance, national guidelines for CICT mentioned mobilization of public health professionals, nurse/paramedics and lab technicians/lab assistants for CICT which is not possible at province and local levels because such human resources are not easily available. Local administrations therefore had to adapt guidelines and make them specific to their context. This was the case, for example, of guidelines for isolation centres and operating procedures regarding CICT.

#### Gender and equity in policies and response measures

Our policy review revealed that gender and equity considerations were not generally reflected in COVID-19 policies and guidelines. A federal level informant confirmed that often gender and equity parameters in policies and guidelines were overlooked as the focus was on finding ways to respond to the emergency situation, rather than considering gender and equity. Similarly, gaps were observed in consideration of gender and equity in provincial level policies and guidelines, especially at the beginning. However, a series of gender related issues started emerging during implementation, for example in relation to quarantine management (common bathing area for males and females, cases of rape etc. occurring in different parts of the country that appeared quite frequently in media sources). This forced a revision of policies at the province level, as a reactive management to inform practices accordingly to the context e.g., separate living and essential health services for children, elderly, pregnant and lactating mothers, people with disability and chronic illnesses in quarantine centres [17], separate room, toilet and bathroom for males and females along with sanitary pads for females, and provision of female security personnel at female isolation centres [18].

*There was no thought about gender [equality and equity] since it was handled based on case. But there were some issues during quarantine management like increased number of people were kept together, both male and females were kept in the same block, bathing area was also same for both male and females in the quarantine centre. However, gender issue was not addressed in policies. [During quarantine management], we witnessed that problem, so we addressed it verbally though it was not mentioned in the policy. Later, the issue had been addressed by arranging separate rooms for male and female. (Province official)*

### Policy communication and information dissemination approaches

The federal government used several channels for policy communication and dissemination. These included for example: daily national press briefings, situation reports, and notices on official websites, social media platforms (Facebook, twitter, Viber), newspapers, local radios, and televisions. Although different mediums were used, the overall communication process was found to be a one-way, top-down approach. Targeted communication to respective audiences was absent as communication to different levels of government, health workers and public was done in the same manner. Interaction for communicating policies between the three tiers of government was largely missing. As a result, provincial and local governments remained less aware of some policies and updates, and thus had to rely on their own access to information.

*There needs to be targeted audience and focused communication. We just did general communication. After making policies, we should have called ministers of all seven provinces, directors and briefed them about the policy. We should have explained the reason for not doing PCR testing after 14 days and explained them about the evidence on which guidelines are based. We did not communicate about it. (MoHP_Federal).*

*There was a communication gap. Federal level formulated the guidelines but never informed us about that. We have to search in Facebook, we knew [about the guidelines] through other mediums. We only operated and managed by exploring [the guidelines] through other mediums and self-search. (Province official)*

In contrast, the provincial government was to some extent more proactively engaging in communicating and updating local governments about new policies, via direct channels such as phone call, email or physical meetings, although that was sluggish in the initial phase. Furthermore, ad-hoc meetings were also conducted between province and local levels for coordination. Later, the provincial government developed a software application that gathered relevant COVID-19 information, national policies and official documents to inform and update local governments and health workers. At the municipal level, municipalities were found to communicate information regarding COVID-19 policies and guidelines to ward representatives and health workers in a simple and comprehensive way, either in person or via phone. They also discussed ways to implement policies and guidelines.

*The federal government did not communicate policies formally. The province government sent model of different format through email. It has also mobilized a responsible person [for communication]. Policies and guidelines keep on changing but the responsible person does the coordination. They call formally and ask us to enter the situation here in that format and we send the data through email. We also take the direction from there. That is how the information is circulated. (Health Coordinator_Municipality1).*

Health workers on the other hand were not officially informed about policies from the municipality or other levels. They were informed verbally by municipality officials but not in any written form or through sharing of documents which they felt to be ineffective and inhibited their understanding. They also relied on their own access to information.

*Regarding the urgent matter like providing vitamin-A during COVID, we got that information through Facebook only… That information should have been forwarded to us, but it was not done. My friends shared it on Facebook and I saw it there. After that, I printed that and shared with the health workers. (Male Health Worker_ Municipality2)*

### Policy implementation

#### Decision space, capacity, and accountability in policy implementation at subnational level

Decision space includes authority and choices to make decisions at the local level, accompanied by strengthened capacities and accountability mechanism for better decision making [19]. Federalism has meant that increased power is assigned to subnational governments, and local governments have *de jure* decision spaces for the operational aspect of disease management such as planning, budgeting, resource allocation for COVID-19 management. In practice, provincial and local governments were found to be exercising this power mostly at the operational level and in terms of policy implementation (rather than in terms of policy formulation), by allocating budget for COVID-19 management, establishing isolation and quarantine centres, procuring equipment and materials (personal protective equipment, masks, sanitizers, soap) and hiring health workers, among other activities. Local governments did not always follow federal guidelines. For example, the guideline for testing was amended by federal government which required no testing after completion of 14 days of isolation. This guideline was not followed by the local governments, and they continued testing for all COVID-19 infected people completing a quarantine period. Moreover, the guidance of the federal government to not allow the migrant population to enter the country through border entry points because of increased risks of transmission of COVID-19 infection was again not followed by local governments. Instead, both local governments included in our analysis permitted entry for migrants who were stranded on the India-Nepal border and placed them in quarantine centres.

However, issues were raised because of the lack of budget available to local governments due to delay in allocation from federal level or the absence of emergency budgets to cover for such a pandemic situation. This highlighted the fact that a large amount of budget remains with federal government despite the decentralization in the country. In addition, respondents noted that duplication of budget happened in some places while budget was insufficient in others. This created confusion and difficulties in policy implementation.

*There was a controversy. Sometimes, federal government directly provided budget for quarantine management to municipal level, whereas sometimes, budget was sent to province and province sent budget to municipal level for quarantine management. Federal, provincial and local government separated budget for quarantine and isolation. It was not clear who should allocate what amount of budget and their exact roles, particularly in the context of COVID 19 response. (EDP_ Province).*

As a result, the overall implementation process was not smooth and, in some instances, resulted in suboptimal implementation. One clear example concerns the management of quarantine and isolation centres where the local governments faced a hard time to establish and manage them in the community. Most quarantine centres were established at schools, community halls, hotels and other spaces, which was not sufficient to quarantine thousands of people entering Nepal from India and other countries. Due to the tremendous load of people in quarantine centres, municipalities were unable to properly manage the quarantine centres and people faced many difficulties in terms of getting quality food, space, privacy and safety, which also affected the safety of the health workforce from the COVID-19 infection.

#### Policy compliance and mechanism for monitoring

Despite the availability of decision space concerning operational mechanisms, a number of respondents at municipal level noted the lack of clarity on the roles and responsibilities of local governments with regard to federal policies, which hindered rapid and effective implementation. This was also confirmed by respondents from federal government. One example concerned the mobilisation of the budget and human resources needed for CICT, which affected the contact tracing and case investigation activities.

*The CICT structure that was [supposed to be] formed all-round the nation was not activated adequately. There was uncertainty regarding who will offer the budget necessary for training, how the training will be conducted. Municipalities were not clear how to manage budget and from where to manage health personnel to form CICT team. (EDP_Federal).*

Policy compliance was challenging due to the sudden changes over which local governments seemed to have no control. For example, respondents at local level recounted how they had tried to build up structures to implement a policy on mobilization of volunteers, only to see it later revised.

*We received a national directive on how to form a volunteer team during the COVID-19 pandemic. Later, after we took a decision and formed a team, we again received another letter from the government due to which we cancelled the mobilization of volunteer teams. (Health Coordinator_Municipality 1).*

A number of mechanisms were put in place by the different levels to monitor compliance to the COVID-19 policies. The federal government recruited and deployed provincial coordinators in order to assess the need of health infrastructures and human resources required for responding to the pandemic and the MoHP at central level made visits to the provincial dedicated hospital and laboratories for monitoring. At provincial level, the Ministry of Health (then functioning under Ministry of Social Development) and PHD along with WHO conducted monitoring and supervision of COVID-19 hospitals, quarantine centres and border entry points, mostly on an ad-hoc basis rather than regularly. Similarly, with support from WHO and UNICEF, the province created “isolation centre joint monitoring teams” to monitor delivery of services and maintenance of standards at quarantine and isolation centres at local levels with the use of a monitoring checklist.

However, neither federal nor provincial levels were able to monitor policy implementation and compliance at community level. The urban municipality included in this study received only one monitoring visit during the entire COVID-19 pandemic. The rural municipality included in this study (but not the urban one) formed ward committees as directed by federal government to monitor policy compliance in terms of self-quarantine, institutional quarantine, and adoption of public health standards (such as, social distancing, wearing mask and sanitizing/washing hands).

## Discussion

This study explored the health sector policy response to COVID-19 in the federalised context of Nepal, highlighting the critical role of the health system in policy formulation, communication and implementation across multiple levels. The study assessed how the response mobilised potential or existing health system resilience capacities and how this has affected its effectiveness, and highlighted areas which require urgent action to build a resilient health system. The findings also demonstrated how different components of the resilience framework [6] interacted that are crucial in building health system resilience. Table 3 provides a summary overview of the main findings aligning with the components of the framework.

**Table 3:** Summary of major findings

Response measures	Challenges
**Emergency planning and policy development** • COVID-19 policy formulation process commenced from March 2020 • About ninety policies and guidelines were developed in the year 2020	**Lack of inclusive and equitable planning and governance** • Active participation of provincial and local government during policy formulation was limited • Gender and equity not well considered in policy formulation, however, reactive management done when related issues started to get reported
**Strategic use of evidence** • Policies guided by global evidence and learnings • Global guidelines actively reviewed and updated at federal level	**Inadequate monitoring of the local context and needs; and lack of mechanisms to ensure accountability** • Policies developed at federal level were not feasible in local contexts • No established mechanism to monitor and ensure policy compliance
**Dedicated leadership and distributed control; multi-sectoral collaboration and networks** • Federal government led policy formulation, with technical leadership from MoHP and CCMC • Formation of committees and groups to respond COVID-19 at different tiers - strong multisectoral collaboration and partnership established at federal level • Use of decision space by provincial government to manage the pandemic response in the areas such as budget allocation, logistics procurement, human resource recruitment, etc.	**Inadequate capacity of system stakeholders and health workers** • Local governments mostly reliant on policies from federal and provincial government • Local adaptation of policies was rarely done due to lack of capacity and experience • Few examples of reactive adaptation of federal and provincial policies into local context, at municipality and health facilities
**Information dissemination and policy communication** • Intensive use of various media channels by federal government such as press briefing, national websites, social medias, newspapers, radios and television to communicate COVID-19 policies and COVID-19 information to all • Use of phone call, email, and meetings to convey information at subnational levels	**Information dissemination approaches not effective and targeted** • Top-down approach to communication • Policy communications not targeted, same information and channels to communicate to diverse audiences– subnational policy makers, health managers, health workers and public - which was found to be ineffective • Lack of clarity on how to implement policy decisions among local government officials and health workers • Sudden changes in policies dictated from higher levels without timely communication

Our findings highlighted that the federal government, who is mainly responsible for the emergency management in the federalised context, effectively handled the overall policy development process with technical leadership from MoHP and CCMC, and by engaging multi-sectoral actors. This fits within the roles in the decentralised context where federal government sets policy and leads management in emergency situation, and local governments translate them into actions with necessary adaptations. However, the participatory and inclusive process of policy formulation with the involvement of other tiers of governments (province and municipalities) and the communities was often ignored. Policies developed at federal level lacked feasibility and applicability in local contexts, which was also highlighted in another study conducted in Nepal [20]. The emergency plans and structures that were established before the federal system in the country were used in the pandemic response, but proved difficult to adapt and implement in varied local settings. Furthermore, the existing community structures at local levels, that are linking communities to the health system were not properly utilised during pandemic response, and thus emphasis should be given to community engagement by sufficiently training and mobilising community health workers (FCHVs including health workers at peripheral level) for the emergency response [21].

Effective coordination and communication was another area for which the federal government was largely criticized. Although various channels and media were used aggressively in communicating COVID-19 policies and information from federal to subnational governments, including to the public, they all used a top-down approach that raised concerns about clarity and understanding of the messages at different layers. The policy communication process, which was not systematic, timely and targeted, resulted in misunderstanding of the decisions and confusion in their implementation at the ground level, where there was no mechanism to monitor and ensure compliance to those policy decisions. Due to lack of clarity in roles and decisions, further delays in action and poor implementation were the resulting consequences. Therefore, crucial to health system resilience is to ensure that the coordination and communication channels and approaches should be appropriate and reach targeted audiences on time with clear messages [22]. Gender and equity was an area that received less attention in the policy documents, nevertheless, reactive management during implementation led to adaptations when issues started to be reported.

Another key area identified in our synthesis was availability of decision space at the local level and the capacity to use it. Local governments, despite having decision space to develop local policies and guidelines in the federalised context, were mainly relying on policies and decisions communicated by provincial and federal governments and their contextual tailoring was very rare. This was due to lack of capacity and experience, and the absence of mechanisms to develop and monitor accountability. Insufficient capacity of local government in decentralised contexts to function appeared to be a common problem across six countries in fragile and conflict-affected settings (namely Pakistan, Philippines, Indonesia, Myanmar, Nepal, Sri Lanka, and Papua New Guinea), as highlighted in a recent study [23]. This capacity gap and the contexts impact the performance, equity and ultimately, resilience of the health system [24]. The response mechanisms implemented at local levels therefore were ad hoc without effective use of evidence and resources [5, 25]. As a consequence, the issue of low trust in local governments among the public remained, which was also highlighted in another study conducted in Cameroon, Nepal and South Africa, where governments struggled to build credibility and acceptance of public during COVID-19 [5]. Moreover, decentralization was seen in administrative structure and functions, while financial control was still centralized (the federal government holds 82% of the programme budget [26]). In line with the context in Nepal, the national level in Myanmar retains control over financing, legislation and the formulation of national policies and plans [27]. The central government in Indonesia holds 90% of the resources where districts have control over only one-third of the total public expenditure on health [23]. A combination of centralization, in which the federal government takes the lead in coordinating and providing policy guidance for improved performance, along with decentralization, where local governments have increased flexibility and decision space to increase equity and resilience [23], is sometimes argued as an effective model of decentralization. This was highlighted in a study in India, where lessons from the responses of individual states’ during COVID-19 suggested that empowering state governments to handle pandemics, while the central government focuses on designing effective strategies, increasing funding and strengthening monitoring mechanisms, can be highly effective [28].

By applying the health system resilience framework [6] to the study findings, our analysis highlights the role played by the resilience capacities of the health system and how they were mobilised in supporting the response to the COVID-19 pandemic. We found that some elements that might have contributed to the resilience of the health system and its capacity to absorb, adapt and transform in the face of the pandemic were already present and were effectively exploited. These were found in particular at federal level and included the rapid and comprehensive activation of already existing emergency structure and plans, which was yet to be tested in the federalised context. In the newly established federal system, such capacity to formulate response policies were meant to be transferred to local levels, alongside the skills and resources necessary to make efficient use of the policy formulation decision space. However, due to the novelty of the federal system and the consequent lack of experience, confusion about roles and responsibilities, insufficient local health system governance and low availability of human, technical and financial resources, those same resilience capacities were not effectively mobilised at local levels. As a consequence, a rapid response, reverting to a pre-federalisation, top-down model prevailed over a participatory approach of inclusive and open governance in decision making, that would have strengthened potentially existing resilience capacities at local levels, or to build them to ensure the longer term resilience of the local health systems. This approach promoted absorption strategies in order to cope with the pandemic but did not generally support adaptive responses of the health system.

Importantly, this last point does not mean that adaptations did not occur at the local level. While policies formulated at central level were reflecting less on local contexts and needs and did not build on locally relevant intelligence and data (rather built on global evidence), local governments were able to partially take advantage of the decision space allowed by the federalization process, at least in terms of policy communication approaches and policy implementation. We found a few examples of local tailoring of policies and guidelines, although often this was reactive rather than proactive and hampered by lack of financial, human and material resources, ambiguity in roles and responsibilities, and lack of capacity for information gathering and implementation monitoring. For example, the local governments mobilized the CICT team, adapting to the local context due to the unavailability of technical human resources indicated in the guideline. Likewise, adaptation was also seen in the operation of quarantine and isolation centres for management of COVID-19 cases.

### Strengths and limitations of the study

This study aimed to explore health system resilience in Nepal in responding to COVID-19 pandemic, taking examples from two municipalities as a case study. The study offers some important insights on the need for an inclusive policy formulation process and effective communication strategies which use the right channels and approaches to reach targeted audiences in a decentralized context. These are crucial components of pandemic responses but less emphasized in other studies. Moreover, the study also shares some experiences of the country in the transition to federalization around coordination and partnership, translating capacities to local governments and generating local leadership, accountability and trust, which are instrumental to strengthen and build resilient health systems across multiple levels. However, there are some limitations to our work. The findings of this study may not be representative of wider contexts, and thus demand further research of a larger scale. However, the coping mechanisms adopted and the resilient capacities shown by the country and the local governments, and the key lessons learnt, generate important learnings for the country itself, and for other similar settings to consider during future shocks and emergencies.

## Conclusions

This study has assessed key resilience capacities of the health system required to manage shocks, such as COVID-19 pandemic. It is clear that a strong and flexible command structure is essential in effectively dealing with an emergency situation. Although the federal government has a key role in emergency response, there is a need for decentralized frameworks to be used in emergency situations, where strengthening capacity of local governments is one of the key areas of focus, in addition to investment in infrastructure and equipment. Inclusive, responsive, evidence- and needs-based, and gender equitable policies and adoption of a clear and effective approaches to communicate the policies are crucial to building resilience to protect population health in the situation of emergency and changing health needs. Continued learning and adaptation from the COVID-19 pandemic, and from other events of acute and chronic shocks to the health system in countries undergoing structural transitions will help build resilience in the long run.

### Electronic supplementary material

Below is the link to the electronic supplementary material.

Supplementary Material 1

## Data Availability

The data generated and analysed from KIIs in this study are not publicly available because they contain interviews from informants who consented for use of the data in this study and thus would not allow for public storage. Data are available from corresponding author on request.

## Abbreviations

CCMC: COVID-19 Crisis Management Center
CICT: Case Investigation and Contact Tracing
COVID-19: Coronavirus Disease
DoHS: Department of Health Services
EDCD: Epidemiology and Disease Control Division
EDP: External Development Partner
FCHV: Female Community Health Volunteer
HEOC: Health Emergency Operation Center
KIIs: Key Informant Interviews
MoFAGA: Ministry of Federal Affairs and General Administration
MoHP: Ministry of Health and Population
NGO: Non-Governmental Organization
PHD: Provincial Health Directorate
UNICEF: United Nations Children’s Fund
WHO: World Health Organization

## References

[1] Barasa E, Mbau R, Gilson L. What is resilience and how can it be nurtured? A systematic review of empirical literature on organizational resilience. Int J Heal Policy Manag. 2018;7(6):491–503. 10.15171/ijhpm.2018.06. 10.15171/ijhpm.2018.06 PMC6015506 29935126

[2] Blanchet K, Nam SL, Ramalingam B, Pozo-Martin F. Governance and capacity to manage resilience of health systems: Towards a new conceptual framework. Int J Heal Policy Manag. 2017;6(8):431–5. 10.15171/ijhpm.2017.36. 10.15171/ijhpm.2017.36 PMC5553211 28812842

[3] Barasa EW, Cloete K, Gilson L (2017). From bouncing back, to nurturing emergence: reframing the concept of resilience in health systems strengthening. Health Policy Plan.

[4] Witter S, Thomas S, Topp SM et al. Health system resilience: a critical review and reconceptualisation. 11, The Lancet. Global health. NLM (Medline); 2023. p. e1454–8. 10.1016/S2214-109X(23)00279-6 37591591

[5] Williams G, Thinking, and Working Politically on Health Systems Resilience.: 2022;(June):1–13. Available from: https://twpcommunity.org/wp-content/uploads/2022/06/TWP-about-health-systems-resilience-reflection-note-final.pdf. Accessed 17 November 2022.

[6] Witter S, Diaconu K, Bertone M, Baral S, Fouad F, Than KK, Wurie H, Raven J. ReBUILD for Resilience Framework and Hypotheses. 2020. Available from: https://www.rebuildconsortium.com/research-themes/resilience-framework-2/. Accessed 8 April 2023.

[7] Jamal Z, Alameddine M, Diaconu K (2020). Health system resilience in the face of crisis: analysing the challenges, strategies and capacities for UNRWA in Syria. Health Policy Plan.

[8] Thapa R, Bam K, Tiwari P, Sinha TK, Dahal S. Implementing federalism in the health system of Nepal: Opportunities and challenges. Int J Heal Policy Manag. 2019;8(4):195–8. 10.15171/ijhpm.2018.121. 10.15171/ijhpm.2018.121 PMC6499910 31050964

[9] Vaidya A, Simkhada P, Simkhada B (2020). The Impact of Federalization on Health Sector in Nepal: New opportunities and challenges. J Nepal Health Res Counc.

[10] Mathieu E, Ritchie H, Rodés-Guirao L et al. Coronavirus Pandemic (COVID-19). Our World Data. 2020; Available from: https://ourworldindata.org/coronavirus. Accessed 9 July 2021.

[11] Cucinotta D, Vanelli M, WHO Declares COVID-19 a Pandemic. Acta Biomed. 2020;91:157–60. 10.7326/M20-0504. Accessed 26 April 2023. 10.23750/abm.v91i1.9397 PMC7569573 32191675

[12] Ministry of Health and Population. Responding to CoVid-19: Health sector preparedness, response and lessons learnt. Kathmandu; 2021.

[13] Ministry of Federal Affairs and General Administration (MoFAGA). Regarding Essential Management for Coronavirus (COVID-19) Preparedness and Response (To all local levels). 2020. Available from: https://mofaga.gov.np/news-notice/1795. Accessed 26 November 2020.

[14] Ministry of Federal Affairs and General Administration (MoFAGA). Decision of the Council of Ministers of the Government of Nepal dated 2076.12.16 on the issue related to COVID-19. 2020. Available from: https://mofaga.gov.np/news-notice/1812. Accessed 26 November 2020.

[15] Ghanshyam Gautam K, Khanal T, Bondurant. An analysis of the health sector functions of all three levels of government as per functional analysis and assignments and relevant policies. NHSSP. 2020.

[16] Ministry of Federal Affairs and General Administration (MoFAGA). Regarding implementation of decision of Government of Nepal (To all local levels). 2020. Available from: https://mofaga.gov.np/news-notice/1806. Accessed 26 November 2020.

[17] Ministry of Federal Affairs and General Administration (MoFAGA). Guidelines for operation and management of quarantine. 2020. Available from: https://mofaga.gov.np/news-notice/1803. Accessed 26 November 2020.

[18] Ministry of Health and Population (Government of Nepal). COVID-19 Cases Isolation Management Guidelines. 2020;0–4. Available from: https://publichealthupdate.com/covid-19-cases-isolation-management-guideline/.

[19] Liwanag HJ, Wyss K (2019). Optimising decentralisation for the health sector by exploring the synergy of decision space, capacity and accountability: insights from the Philippines. Heal Res Policy Syst.

[20] Wasti SP, Simkhada P, Ale S, Van Teijlingen E (2021). Nepalese Health System Response to fight against COVID-19 pandemic. Eur J Med Sci.

[21] Haldane V, De Foo C, Abdalla SM et al. Health systems resilience in managing the COVID-19 pandemic: lessons from 28 countries. Nat Med. 2021;27(6):964–80. 10.1038/s41591-021-01381-y. 10.1038/s41591-021-01381-y 34002090

[22] Gooding K, Bertone MP, Loffreda G, Witter S. How can we strengthen partnership and coordination for health system emergency preparedness and response? Findings from a synthesis of experience across countries facing shocks. BMC Health Serv Res. 2022;22(1):1–19. 10.1186/s12913-022-08859-6. 10.1186/s12913-022-08859-6 PMC9706990 36447261

[23] Brennan E, Abimbola S. The impact of decentralisation on health systems in fragile and post-conflict countries: a narrative synthesis of six case studies in the Indo-Pacific. Confl Health. 2023;17(31). 10.1186/s13031-023-00528-7 PMC10280898 37340483

[24] Abimbola S, Baatiema L, Bigdeli M. The impacts of decentralization on health system equity, efficiency and resilience: a realist synthesis of the evidence. Health Policy and Planning. Volume 34. Oxford University Press; 2019. pp. 605–17. 10.1093/heapol/czz055 PMC6794566 31378811

[25] Shrestha N, Mishra SR, Ghimire S et al. Health system preparedness for COVID-19 and its impacts on frontline health care workers in Nepal: a qualitative study among frontline healthcare workers and policymakers. Disaster Med Public Health Prep 2021; Jun 18:1–9. 10.1017/dmp.2021.204. 10.1017/dmp.2021.204 PMC8376855 34140051

[26] Ministry of Health and Population, UKaid/NHSSP. Budget Analysis of Health Sector. 2020. Available from: http://www.nhssp.org.np/Resources/PPFM/Budget Analysis of Health Sector FY 2020-21.pdf. Accessed 15 February 2023.

[27] Brennan E, Abimbola S (2020). Understanding and progressing health system decentralisation in Myanmar. Glob Secur - Heal Sci Policy.

[28] Shringare A, Fernandes S (2020). COVID-19 pandemic in India points to need for a decentralized response. State Local Gov Rev.

