# Supplementary material for: Understanding health system resilience in responding to COVID-19 pandemic: experiences and lessons from an evolving context of federalization in Nepal
Source: BMC Health Serv Res. 2024 Apr 4;24:428. doi: 10.1186/s12913-024-10755-0 (PMC10996157; doi:10.1186/s12913-024-10755-0)
Supplement: Supplementary file 1 — Supplementary Material 1 [file 12913_2024_10755_MOESM1_ESM.docx]

**Topic guide**

Key informant interviews with policy makers, planners, health workers, EDPs and technical experts at federal, provincial and local levels

**Objectives:**

1. To explore the process of health sector’s COVID-19 related policy formulation, communication and implementation against the element’s of R4R resilience framework
2. To understand the health workforce availability, capacity and motivation management mechanisms adopted at the national and subnational level to implement COVID-19 response, while ensuring delivery of and access to quality routine health care services
3. To explore key informant’s perceptions of current health sector’s efforts, its strengths and weaknesses in the COVID-19 response and management

**Introduction:** Introduce the project, the scope of the interview

**Informed Consent Process:** Ensure participant has read the information sheet/or information sheet has been read out to the participant, ask if she / he has any questions or areas for clarification, explain about confidentiality including recording the interview and voluntary consent to participate in the interview, complete consent sheet.

| **Information about the interviewee** | | | |
| --- | --- | --- | --- |
| Interviewee ID |  | Job title and cadre of interviewee |  |
| Age |  | Gender | Male □ Female □ Other □ |
| Place of work (organization name) |  | No of years working in this organization |  |
| Years of experience working in health sector |  | Province |  |
| District |  | Municipality |  |
| Name of interviewer |  | Date of Interview |  |
| Time of start of interview |  | Time of end of interview |  |

**Key informant background**

1. What is your current role in the health system?
2. How are you involved in the COVID-19 response? In what capacity?

**Formulation of COVID-19 policies and guidelines**

1. Can you please explain the overall COVID-19 management and response structure at MoHP?
   1. Who prepares policies and guidelines? Who leads the process?
   2. Who are the main actors? (divisions within MoHP, EDPs, technical experts to MoHP etc)
2. What is the process of formulation of policies and plans in response to COVID-19?
   1. Can you please provide examples of HRH related policies?
3. What are the different HRH related policies or guidelines that have been put in place so far?
4. What factors are being considered for HRH related policy formulation? *Probe*: if the policies are evidence-informed, adapted from global learnings, etc.
5. How is the inter-sectoral engagement achieved for HRH policy formulation? *Probe:* different sectors and ministries beyond health and involvement of private sectors, etc)
   1. Who were involved and what are their roles? *Probe* for vertical and horizontal engagement.
   2. What coordination mechanism is in place for involving stakeholders in the formulation of policies?
   3. What challenges have you faced in this engagement process?
6. Are provincial and local governments involved in policy formulation at federal level?
   1. What are their roles in the policy formulation process?
7. Can you please explain how are HRH related policies in the context of COVID-19 formed at provincial and local levels?
   1. Are they guided by national policies/guidelines?
   2. Sub-nationals only: Are the national policies applicable in the local context? Please share your experience.
8. Are any gender or equity related considerations reflected in any COVID-19 related policies or guidelines?
   1. How are these reflected in HRH related policies and guidelines?

**Coordination and policy communication**

1. Can you please describe how the three tiers of government interact for policy communication?
   1. How are policies communicated to the different tiers of government?
   2. Do you think there is clarity on roles and coordination mechanisms at all levels of government? Why?
   3. What are the challenges you have faced in this process?
2. What are the communication strategies being used to inform and update health staff at all levels?
   1. What are the channels used? (including formal and informal platforms, such as Viber groups, Apps, etc.).
   2. What are the coordination mechanisms to ensure consistency of the information supplied to health staff (eg. on specific risks and self-protection measures)?
3. Is there any mechanism to share day-to-day lessons learned at different levels of the system and from international experience? What are the mechanisms in place?
4. What are the communication strategies in place to inform the public and the media?
   1. Do you think the communication strategies also target minority and vulnerable populations? How?

**Implementation of policies and plans**

1. What is the decision space allowed for at sub-national levels? (In terms of flexibility of budget and spending procedures to respond rapidly to shortages of key supplies, or in terms of staff deployment)
   1. Is anything being done to increase or decrease decision space of local managers? What is being done?
2. What are the mechanisms in place to monitor if different policies and guidelines are being implemented?
   1. Who is responsible for monitoring the compliance at all three levels?
3. What is the routine process of reporting the implementation status of COVID-19 response, and in particular HRH related policies and response?

**Availability, capacity and motivation of HRH**

1. How are HRH managed for the delivery of COVID-19 and non-COVID health services? How is the situation throughout the country?
   1. Is the available number of HRH sufficient to deal with the current COVID-19 situation?
   2. What are the current issues related with availability and skills mix of human resources?
   3. Were the health workers transferred, redeployed to manage the shortages and maintain balance in the availability at different levels?
   4. Are HRH from private sectors redeployed? What is the overall process of mobilization of HR from private sectors?
2. Were the health workers trained or oriented on IPC and safety measures for delivery of services in COVID-19 context?
   1. Are all the health workforce currently mobilized in COVID-19 services trained/oriented?
   2. What kind of training were provided to different cadres of health workers?
   3. Who provided the training to health workforce at different levels?
3. What has been done to protect the physical health of frontline health workers?
   1. What types of PPE are being provided? Are the PPE sufficient? What is being done for uninterrupted supply of required PPE?
4. What are being done to support mental health of frontline health workers?
5. How is health staff being supported, recognized and encouraged?
   1. What are the provisions in place for motivation or incentives to human resources involved in COVID-19 prevention and treatment? (eg, risk allowance, insurance etc)
   2. Is the any mechanism for supportive supervision? Who is involved in supportive supervision and which cadre of HR are monitored and supervised?
6. How are the working hours of human resources being managed?
   1. Are staff having to work longer hours?
   2. What is the leave or holiday provision for them?
   3. Are any gender related consideration adapted during mobilization of human resources in COVID-19 prevention and treatment? What are those considerations?
7. How are female community health volunteers (FCHVs) mobilized in preparedness and response activities in the given context? What are the assigned responsibilities in the present context?
   1. How is their safety ensured? What personal protective equipment have they received from government?
   2. Have they received any training/orientation for working in current context?
   3. What are the incentives or benefits provision to FCHVs for working in this context?

**Recommendation/lessons learnt**

1. How well do you think the health system has been able to manage the COVID pandemic so far?
   1. What do you see as the main strengths of health system in coping with the shock?
   2. What are the major weaknesses?
2. From the learnings of COVID-19, what could be done, within existing resources, to improve the response, recovery and preparedness for the next shock?

END, THANK YOU !
